# The Measles Virus Receptor SLAMF1 Can Mediate Particle Endocytosis

**DOI:** 10.1128/JVI.02255-16

**Published:** 2017-03-13

**Authors:** Daniel Gonçalves-Carneiro, Jane A. McKeating, Dalan Bailey

**Affiliations:** a Centre for Human Virology, Institute of Immunology and Immunotherapy, College of Medical and Dental Sciences, University of Birmingham, Birmingham, United Kingdom; b Institute for Advanced Study, Technische Universität München, Munich, Germany; University of Iowa

**Keywords:** SLAMF1, endocytosis, fusion, macropinocytosis, measles, morbillivirus, virus attachment, virus entry

## Abstract

The signaling lymphocyte activation molecule F1 (SLAMF1) is both a microbial sensor and entry receptor for measles virus (MeV). Herein, we describe a new role for SLAMF1 to mediate MeV endocytosis that is in contrast with the alternative, and generally accepted, model that MeV genome enters cells only after fusion at the cell surface. We demonstrated that MeV engagement of SLAMF1 induces dramatic but transient morphological changes, most prominently in the formation of membrane blebs, which were shown to colocalize with incoming viral particles, and rearrangement of the actin cytoskeleton in infected cells. MeV infection was dependent on these dynamic cytoskeletal changes as well as fluid uptake through a macropinocytosis-like pathway as chemical inhibition of these processes inhibited entry. Moreover, we identified a role for the RhoA-ROCK-myosin II signaling axis in this MeV internalization process, highlighting a novel role for this recently characterized pathway in virus entry. Our study shows that MeV can hijack a microbial sensor normally involved in bacterial phagocytosis to drive endocytosis using a complex pathway that shares features with canonical viral macropinocytosis, phagocytosis, and mechanotransduction. This uptake pathway is specific to SLAMF1-positive cells and occurs within 60 min of viral attachment. Measles virus remains a significant cause of mortality in human populations, and this research sheds new light on the very first steps of infection of this important pathogen.

**IMPORTANCE** Measles is a significant disease in humans and is estimated to have killed over 200 million people since records began. According to current World Health Organization statistics, it still kills over 100,000 people a year, mostly children in the developing world. The causative agent, measles virus, is a small enveloped RNA virus that infects a broad range of cells during infection. In particular, immune cells are infected via interactions between glycoproteins found on the surface of the virus and SLAMF1, the immune cell receptor. In this study, we have investigated the steps governing entry of measles virus into SLAMF1-positive cells and identified endocytic uptake of viral particles. This research will impact our understanding of morbillivirus-related immunosuppression as well as the application of measles virus as an oncolytic therapeutic.

## INTRODUCTION

The signaling lymphocyte activation molecule family (SLAMF) comprises nine cell surface-expressed glycoproteins that are involved in immune cell interactions and belong to the immunoglobulin superfamily (1). In particular, SLAMF1 (also known as CD150) is expressed on activated B and T lymphocytes as well as dendritic cells and macrophages, and it has been implicated in lymphocyte development, thymocyte maturation, and immunological synapse formation (2, 3). Of note, SLAMF1 was recently reported to act as a microbial sensor and to regulate host immune responses to diverse bacteria (4). SLAMF1 binds outer membrane porins F and C, expressed by Gram-negative bacteria, and stimulates the recruitment of autophagic components via a beclin-1-, Vps34-, and Vps15-dependent pathway, leading to phagocytic killing of the microbe (4).

In contrast to the antimicrobial function, SLAMF1 is the major entry receptor for measles virus (MeV) (5). As recently as 1980, MeV was estimated to have caused 2.6 million deaths per year; however, routine vaccination has now brought this figure down to 100,000. In humans, inhalation of virus-contaminated aerosols leads to MeV infection of macrophages and dendritic cells within the lungs (6). Infection spreads to the local lymph nodes, where a range of SLAMF1-expressing immune cells become infected. The subsequent circulation of MeV-infected SLAMF1^+^ cells in the periphery leads to infection of epithelial tissues in the respiratory tract, which facilitates transmission to new hosts (6, 7). Infection of these epithelial cells is facilitated by a separate receptor, nectin-4, a component of the adherens junction (8, 9). MeV spread from immune cells to nectin-4-positive epithelia is dependent on cell-cell contact, consistent with the highly cell-associated nature of MeV infection (10). As well as delivering MeV to sites for onward transmission, infection of SLAMF1^+^ lymphocytes and monocytes also contributes to the prolonged immunosuppression reported for infected subjects and may explain their increased susceptibility to secondary bacterial infections.

MeV encodes two viral glycoproteins, the hemagglutinin (H) and fusion (F) proteins, that mediate particle entry. Virus-associated H interacts with SLAMF1 and induces conformational changes that activate F (11, 12) and prime fusion of the viral and host cell membranes (13, 14). In addition, F and H induce the fusion of infected and naive cells at neutral pH, leading to multinucleated cells or syncytia (15). This widely reported phenomenon has led to the assumption that MeV enters cells only through direct fusion with the cellular plasma membrane, with a limited requirement for particle endocytosis (16); however, and importantly, there is no definitive study that demonstrates that this phenomenon is the only mechanism for entry. Building on the identification of SLAMF1 as a sensor involved in bacterial phagocytosis along with recent studies reporting on the endocytosis of related viruses, such as Ebola virus and respiratory syncytial virus (RSV) (17, 18), we revisited the pathway of MeV entry. Herein, we describe a novel internalization pathway for MeV to establish infection that is dependent on its immune receptor, SLAMF1. Understanding the complex role of SLAMF1-microbe interactions will impact our knowledge of innate immunity, immunosuppression, and host-pathogen interactions.

## RESULTS

### MeV induces the formation of transient membrane structures.

**FIG 1:**
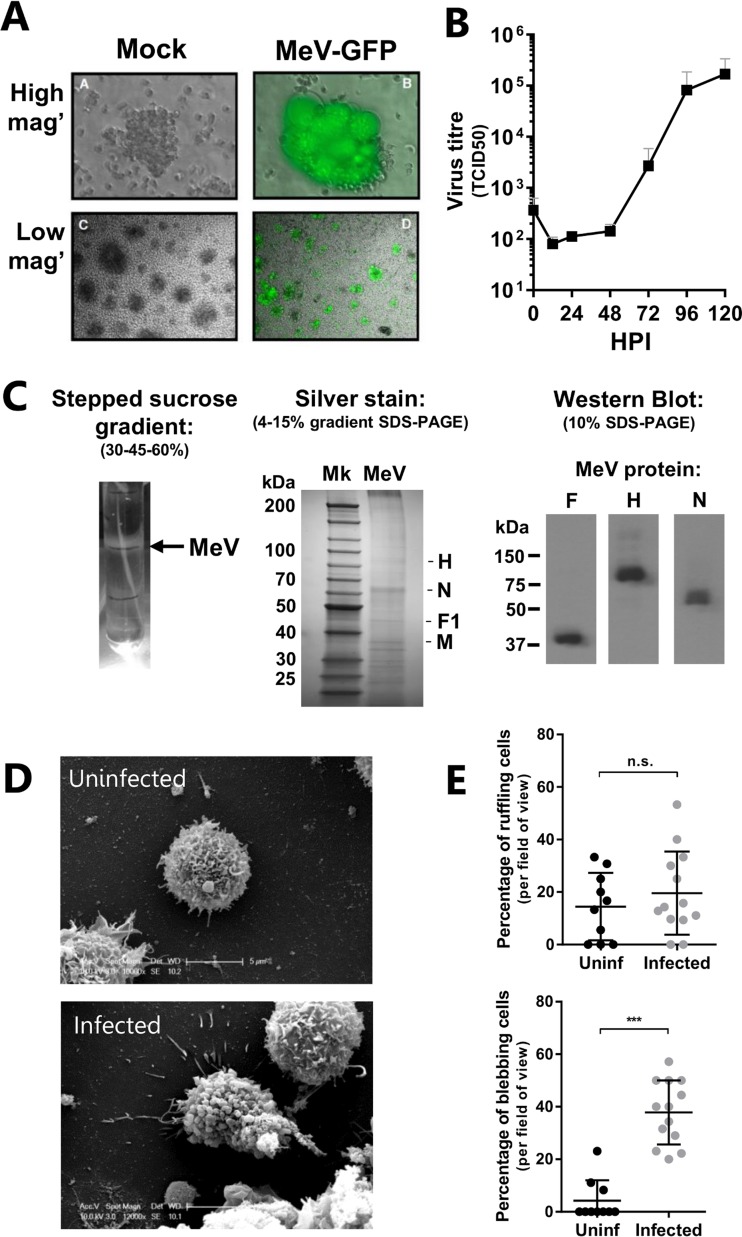
MeV induces the formation of transient membrane structures in SLAMF1-positive lymphocytes. (A) B-lymphoblastoid cells were inoculated with recombinant MeV (MOI, ∼0.1) engineered to express EGFP and incubated for 72 h prior to visualization by UV phase-contrast microscopy at high and low magnifications (mag'). (B) B-lymphoblastoid cells were infected with MeV (MOI, ∼0.01) and incubated at 37°C prior to quantification of viral progeny, by 50% tissue culture infective dose (TCID_50_), at the indicated times. HPI, hours postinfection. (C) MeV was purified by ultracentrifugation using a stepped sucrose gradient. The opalescent band located at 30-45% sucrose cushion interface was extracted and dialyzed. Protein lysates from these purified virus preparations were analyzed by silver staining and Western blot analysis. Mk, protein marker. (D) B-lymphoblastoid cells were synchronously infected with MeV (MOI, ∼20) or mock infected with 20% FBS-containing DMEM before fixation, preparation, and visualization by SEM. (E) Images were blinded and representative micrographs quantified for membrane blebs and membrane ruffles. Statistical analysis was performed using the Student *t* test. ***, *P* < 0.001. n.s., nonsignificant.

Internalization pathways such as phagocytosis and macropinocytosis involve a rearrangement of the plasma membrane (19), with membrane blebbing associated with the uptake of several pathogens, such as Pseudomonas aeruginosa (20) and vaccinia virus (21). We studied the effect of MeV interaction with a patient-derived B-lymphoblastoid cell line that expresses SLAMF1 (6). These cells support MeV replication and generate substantial levels of infectious progeny (Fig. 1A and B). To examine the response to infection, B-lymphoblastoid cells were synchronously infected with purified MeV particles (the cellular contaminants having been removed by sucrose gradient-based ultracentrifugation [Fig. 1C]), through initial adsorption at 4°C for 1 h. Particle purity was assessed by silver staining and Western blotting of virus preparations (Fig. 1C). A high multiplicity of infection (MOI) was used to identify and observe wholescale physiological responses to infection by microscopy, in accordance with previous studies (17). Unbound virus was removed by washing, and the cells were incubated at 37°C for 20 min before examination by scanning electron microscopy (SEM) (Fig. 1D). Infected B-lymphoblastoid cells showed significantly more membrane blebs than uninfected cells (*P* < 0.005; *t* test) (Fig. 1E, bottom graph); however, the frequency of cells exhibiting membrane ruffles did not vary (Fig. 1E, top graph).

**FIG 2:**
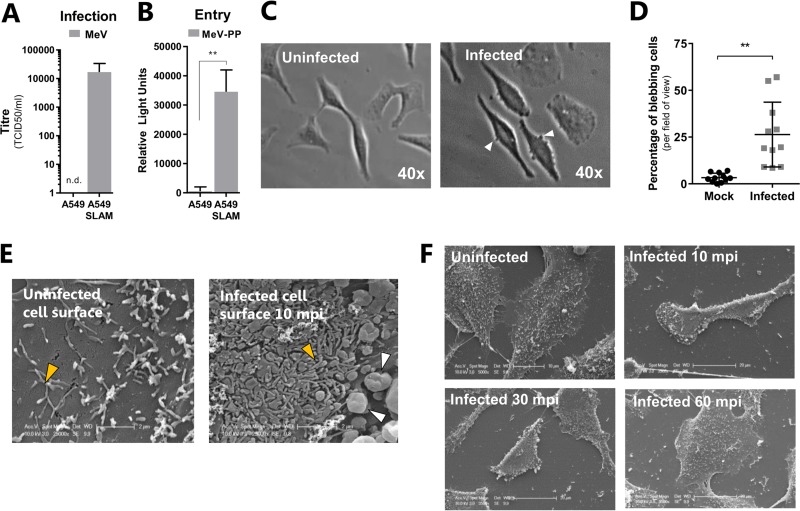
MeV infection of SLAMF1-recombinant A549 cells induces blebbing, filopodium formation, and cellular retraction. (A) A549 or A549-SLAMF1 cells were infected with MeV (MOI, ∼1) and incubated for 48 h prior to cell lysis, clarification of supernatant, and quantification of viral progeny via TCID_50_ (n.d., not detected). (B) Similarly, A549 and A549-SLAMF1 cells were infected with MeV-PP reporter virus and incubated for 72 h prior to lysis and measurement of luciferase activity. (C and D) A549-SLAM cells were serum starved overnight and inoculated with MeV (MOI = 45) for 1 h at 4°C. Cells were washed with PBS and incubated at 37°C for 30 min before fixation with 4% PFA and visualization by phase-contrast microscopy. Representative micrographs of infected and uninfected cells are provided at a magnification of ×40. (C) The percentage of cells that presented membrane blebs per field of view was calculated for both samples (D). (E and F) Similarly, serum-starved A549-SLAMF1 cells were synchronously infected with MeV (MOI = 30) and incubated at 37°C for 0, 10, 30 or 60 min postinfection (mpi) before fixation and imaging by SEM. The cell surface of uninfected and infected cells presented small filopodium-like structures at the surface (orange arrowheads), but only infected cells presented membrane blebs (white arrowheads) (E). Infected A549-SLAMF1 cells retracted after infection, in association with these membrane blebs; however, this was transient and lasted only 60 min (F). All statistical analysis was performed using the Student *t* test. **, *P* < 0.005; error bars indicate SDs.

To examine the SLAMF1 dependency and cell type specificity of MeV-induced changes in cellular morphology, we generated human lung epithelial A549 cells stably expressing SLAMF1 and demonstrated their ability to support infection with MeV (Fig. 2A) and lentiviral pseudotypes bearing MeV-encoded F and H (MeV-PP) (Fig. 2B), a tool that allows specific and quantitative assessment of MeV entry (22). MeV induced a significant increase in membrane blebs within 20 min (Fig. 2C and D), and SEM of infected SLAMF1 cells identified additional morphological changes, including extensive filopodium formation on the cell surface (Fig. 2E, orange arrowheads) and cellular contraction (Fig. 2F) compared to those of uninfected cells. Interestingly, infection-induced blebbing (Fig. 2E, white arrowheads), filopodium formation, and contraction were transient and resolved by 60 min postinfection (Fig. 2F). These data highlight a conserved SLAMF1-dependent cellular response to MeV infection that exhibits hallmarks of endocytic uptake.

### MeV particles can be endocytosed.

**FIG 3:**
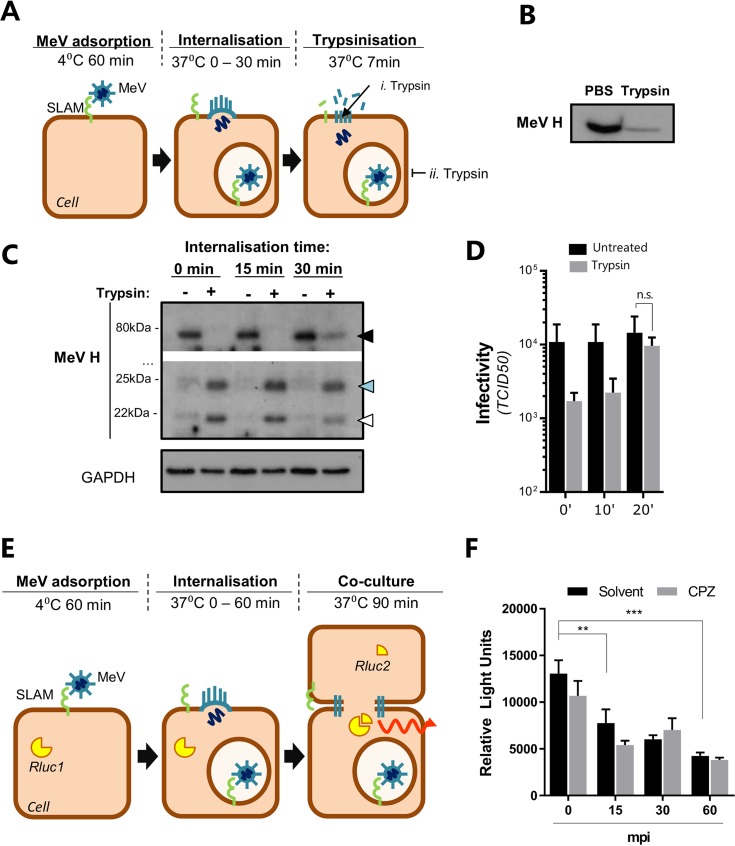
MeV particles are endocytosed. (A) Diagram of experimental setup. Purified MeV was attached to A549-SLAMF1 cells (MOI = 30) at 4°C for 1 h, washed with PBS to remove unbound virus, and incubated for 0, 15, or 30 min at 37°C prior to trypsin treatment. (B) MeV particles were incubated with trypsin at 37°C for 7 min prior to addition of an equal volume of complete fetal calf serum (FCS)-containing cell culture medium. Lysates were generated with Laemmli buffer and resolved on a 15% SDS-PAGE gel, followed by Western blotting with anti-MeV H antibody. (C) Total cell lysates were generated and resolved on a 15% SDS-PAGE gel prior to Western blotting using a polyclonal antibody raised against the cytoplasmic tail of MeV H. Three H-specific bands were detected with approximate sizes of 75 kDa (black arrowhead; full length), 24 kDa (blue arrowhead), and 20 kDa (white arrowhead), the latter two detected only after trypsin treatment. The data are representative of those from four individual experiments. Of note, the specificity of this assay was preserved using an antibody specific to the intravirion tail of MeV H that allowed detection of tryptically digested variants. (D) A549-SLAMF1 cells were synchronously infected with MeV (MOI, ∼1), incubated for the indicated times (minutes postinfection), and trypsinized for 7 min at 37°C. Cells were pelleted, resuspended in complete medium, and incubated for 48 h, and virus infectivity was determined (error bars indicate SDs). (E and F) HEK293T-SLAMF1 cells carrying a split form of Renilla luciferase (RLuc1) were synchronously infected with purified MeV (MOI = 30) for 60 min at 4°C, washed with PBS, and incubated for 0, 15, 30 or 60 min postinfection (mpi), with or without 5 μg/ml of chlorpromazine (CPZ), an inhibitor of clathrin pit formation and membrane protein sequestration, at 37°C prior to the addition of HEK293T-SLAMF1 cells expressing the complementary part of the luciferase (RLuc2). Cells were cocultured for 90 min before addition of the luciferase substrate coelenterazine and measurement of light in a luminometer. Values were normalized by subtracting the values from uninfected cells and plotted as relative light units (error bars indicate standard errors). Statistical analysis was performed using the Student *t* test. **, *P* < 0.005; ***, *P* < 0.001. n.s., nonsignificant.

Although it is generally assumed that MeV can fuse at the plasma membrane, the morphological response we observed following infection of B-lymphoblastoid cells and A549-SLAM cells resembled membrane structures reported during macro- and phagocytosis (23), suggesting that MeV can also be internalized. To investigate this further, we examined the sensitivity of virus-associated H to digestion with trypsin (see schematic in Fig. 3A). We confirmed the sensitivity of MeV-associated glycoproteins to tryptic digestion (Fig. 3B). A549-SLAMF1 cells were synchronously infected, through adsorption at 4°C for 1 h (purified MeV; MOI, ∼30), washed, and incubated at 37°C for 0, 15, or 30 min prior to treatment with trypsin (Fig. 3A). MeV H was detected in all nontrypsinized cells at its full-length size of 75 kDa and was sensitive to trypsin cleavage both immediately after viral adsorption and 15 min later (Fig. 3C, blue and white arrowheads, i. Trypsin). However, when infected cells were incubated at 37°C for longer periods nondigested H was detected, consistent with particle internalization and protection from trypsin (Fig. 3C, black arrowhead, ii. Trypsin). Accordingly, when virus yields from a similar experiment were assayed 48 h postinfection to assess the fate of internalized virus, protection from trypsin equivalent to that of untreated cells was seen (Fig. 3D). To validate these observations, we used an adapted MeV cell-cell fusion assay (13) that relies on cell-bound virus-associated glycoproteins to fuse neighboring cells (see schematic in Fig. 3E). This assay showed a loss in MeV glycoprotein-dependent fusion over time, consistent with particle internalization (Fig. 3F). Importantly, this process was not inhibited by chlorpromazine (CPZ), a drug that blocks the formation of clathrin-coated pits, an important step in the recycling of surface proteins that could feasibly take place after MeV entry at the plasma membrane (Fig. 3F). Together, these data support a model of MeV internalization and suggest multiple pathways for MeV to establish infection.

### MeV internalization is independent of clathrin-, caveolin-, or Dyn2-mediated endocytosis.

**FIG 4:**
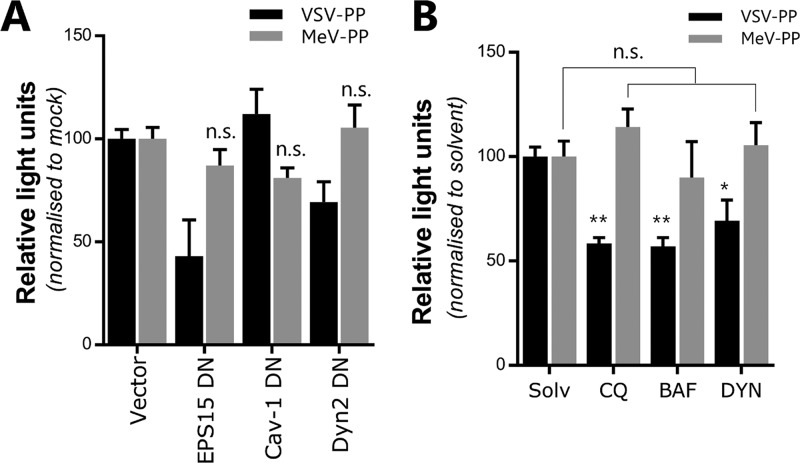
MeV particles are internalized in a clathrin-, caveolin-, and dynamin-2-independent manner. (A) A549-SLAMF1 cells were transfected with plasmids expressing dominant negative (DN) mutants of EPS15, cav-1, and Dyn2 and 48 h posttransfection were transduced with MeV- or VSV-PPs. As a control, cells were transfected with an empty plasmid (Vector). After 72 h, cells were lysed and luciferase activity was measured. (B) A549-SLAMF1 cells were pretreated with chloroquine (CQ; 50 μg/ml), bafilomycin A1 (BAF; 50 μg/ml), dynasore (DYN; 100 μM), or solvent for 30 min at 37°C. MeV- or VSV-PP encoding the firefly luciferase reporter was added to cells and incubated for 3 h. Medium was replaced and cells were incubated for 72 h before lysis, addition of the luciferase substrate luciferin, and measurement of produced light. Statistical analysis was performed using the Student *t* test. *, *P* < 0.05; **, *P* < 0.005. n.s., nonsignificant (error bars indicate standard errors).

To further examine the potential for MeV endocytosis, we employed a quantitative particle entry assay based on reporter HIV-1 pseudotypes bearing MeV F and H (MeV-PP) (22). MeV-PP entry into A549-SLAMF1 cells was not sensitive to green fluorescent protein (GFP)-tagged dominant negative (DN) mutant proteins involved in clathrin-mediated endocytosis (epidermal growth factor receptor pathway substrate 15 [EPS15]), caveolin-mediated endocytosis (caveolin-1 [Cav-1]), or dynamin-dependent endocytosis (dynamin-2 [Dyn2]) (Fig. 4A). In contrast, vesicular stomatitis virus glycoprotein (VSV-G)-pseudotyped HIV-1 (VSV-PP), which is known to enter via clathrin-mediated endocytosis, was sensitive to enhanced GFP (EGFP)-tagged DN EPS15 (Fig. 4A). In all instances, the efficient expression of DN mutants was confirmed through observation of GFP expression. Similarly, chloroquine and bafilomycin A1, lysosomotropic agents that inhibit endosomal acidification and caveolin-dependent endocytosis, respectively, had no effect on MeV-PP entry but reduced VSV-PP infection, as did the dynamin-2 inhibitor dynasore (Fig. 4B). Collectively, these data show that MeV particle internalization is independent of clathrin-, caveolin-, or dynamin-2-mediated endocytic pathways.

### MeV induces fluid uptake in a SLAMF1-dependent manner.

**FIG 5:**
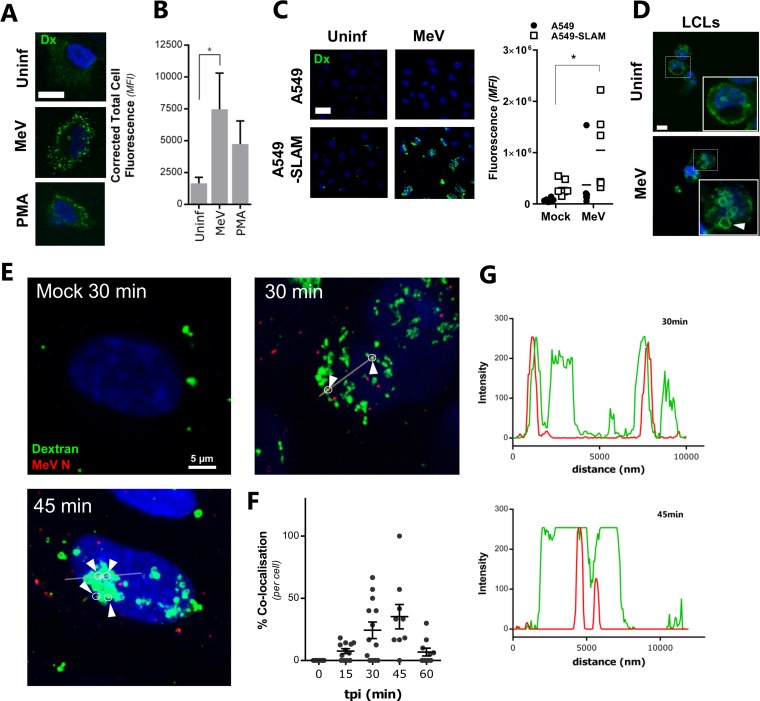
MeV induces fluid uptake in a SLAMF1-dependent manner. (A) A549-SLAMF1 cells were synchronously infected with MeV (MOI = 10) for 1 h at 4°C, washed in cold PBS, and incubated at 37°C for 20 min with DMEM containing 0.25 mg/ml of dextran-Alexa Fluor 488 conjugate. Alternatively, cells were treated with a 200 nM concentration of the macropinocytosis inducer phorbol 12-myristate 13-acetate (PMA). Cells were moved to ice, washed, bleached, and fixed in 4% PFA. Samples were analyzed by CSLM. Representative micrographs of uninfected, MeV-infected, and PMA-treated cells were recorded. The bar represents 15 μm. (B) Quantification of internalized dextran (pseudocolored green) was performed by calculating corrected total cell fluorescence based on mean fluorescence intensity (MFI). Statistical analysis was performed using the Student *t* test. *, *P* < 0.05 (error bars indicate standard errors). (C) To assess the role of SLAMF1 in fluid uptake during MeV infection, A549 and A549-SLAMF1 cells were synchronously mock or MeV infected (MOI = 10), incubated with dextran-containing PBS for 30 min, fixed, and analyzed by CSLM (left) with the mean fluorescent intensity quantified and expressed relative to the background (right). (D) B-lymphoblastoid cells (LCLs) were attached to a glass coverslip, synchronously infected with MeV (MOI, ∼20), and incubated with DMEM containing 0.25 mg/ml of dextran-Alexa Fluor 488 conjugate. Cells were moved to ice, washed, bleached, and fixed before analysis by CSLM. The bar represents 5 μm. (E) A549-SLAMF1 cells were synchronously infected with MeV (MOI = 10), washed and incubated at 37°C in dextran-containing PBS for the indicated times, fixed, prepared, and analyzed by CSLM. Dextran is showed in green, while MeV N is pseudocolored in red; arrowheads indicate colocalization of N and dextran-positive vesicles. (F) Using a similar approach and CSLM analysis, the colocalization of MeV N puncta with dextran-containing endosomes was quantified at 0, 15, 30, 45, and 60 min postinfection. Statistical analysis was performed using analysis of variance (ANOVA); *F* (4, 47; ratio variables obtained with a Brown-Forsythe test) = 6.076; *P* = 0.0005 (error bars indicate standard errors). (G) Intensity analysis was performed using Leiss ZEN software, by profiling of red and green channels along the indicated line.

The internalization of extracellular fluid, assayed through uptake and concentration of cell-impermeative fluorescent dextran, is considered a benchmark for macropinocytosis (23). MeV (MOI, ∼10) and the macropinocytosis-inducing phorbol 12-myristate 13-acetate (PMA) induce fluid uptake and formation of fluorescent dextran-positive vesicles in A549-SLAMF1 and B-lymphoblastoid cells (Fig. 5A to D). Confocal scanning laser microscopy (CSLM) imaging and quantification of the punctate dextran signal in macropinosomes confirmed that MeV induced a significant increase in intracellular dextran (Fig. 5A and B) that was, importantly, SLAMF1 dependent and not seen in nonpermissive parental A549 cells (Fig. 5C) yet was seen in B-lymphoblastoid cells (Fig. 5D). Since we could readily detect dextran-stained vesicles, we next investigated whether these macropinosomes represent sites of internalized MeV. Indeed, we demonstrated that incoming MeV colocalizes with dextran-positive macropinosomes (Fig. 5E). Quantification of this colocalization indicated that this was most prevalent between 30 and 45 min after infection, concurrent with the wide-scale formation of dextran-positive puncta (Fig. 5F). Computational analysis of the fluorescent signals confirmed the specificity of this colocalization as well as the enlargement of dextran-positive macropinosomes (Fig. 5G). Combined, these data suggest that MeV particle and fluid uptake occur concurrently after infection and that this process is SLAMF1 dependent.

### MeV is sensitive to inhibition of macropinocytosis.

**FIG 6:**
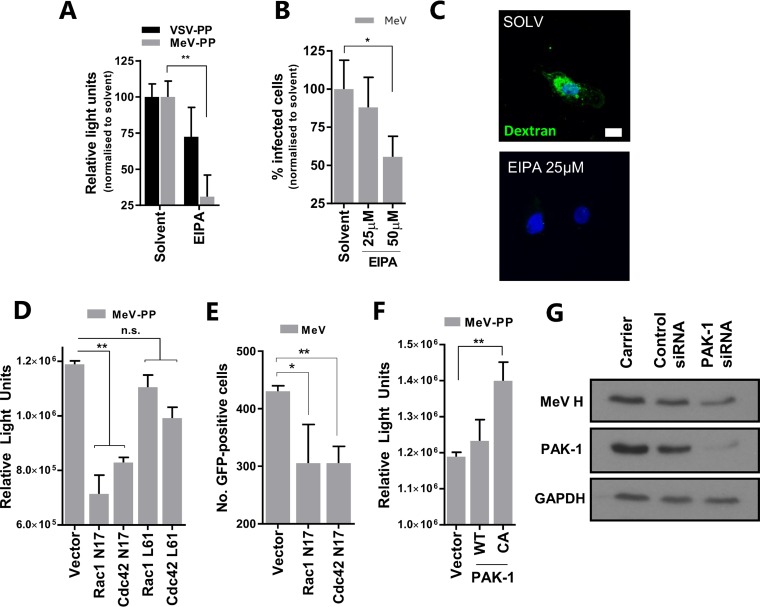
MeV infection is inhibited by amiloride. (A) A549-SLAMF1 cells were treated with 25 μM EIPA prior to infection with MeV- or VSV-PPs for 3 h SLAMF1 and incubation in complete medium. Luciferase activity was measured 72 h later. (B) A549-SLAMF1 cells were pretreated with EIPA and infected with MeV (MOI = 1) at the indicated concentrations, before trypsinization and culture at 37°C for an additional 6.5 h. Cells were detached with 2 mM EDTA solution, and GFP fluorescence was analyzed by flow cytometry. (C) A549-SLAM cells were incubated with EIPA at the indicated concentration for 30 min, washed, and incubated with 200 nM PMA in dextran-containing PBS for 30 min. Cells were then bleached, washed, and prepared for CSLM. (D) A549-SLAMF1 cells were transfected with constructs encoding myc-tagged dominant negative (DN) mutants (Rac1 N17 and Cdc42 N17) or GFP-tagged constitutively active (CA) forms (Rac1 L61 and Cdc42 L61) of the indicated RhoGTPases. Cells were then infected with MeV-PP and luciferase activity was measured 72 h later. (E) Similarly, cells were transfected with the same constructs, infected with MeV (MOI = 1), and incubated for 24 h. Infected cells were quantified by UV microscopy. (F) Cells were transfected with plasmid constructs encoding wild-type (WT) or a CA mutant of PAK-1 and infected with MeV-PP before quantification by luciferase assay. (G) A549-SLAMF1 cells were transfected with either random siRNA (control), siRNA targeting PAK-1, or a carrier. Seventy-two hours later, cells were infected with MeV (MOI = 1), incubated for 24 h, and lysed for Western blot analysis. In all instances the expression of DN, WT, or CA proteins was confirmed by Western blotting or fluorescence microscopy (not shown). Statistical analysis was performed using the Student *t* test. *, *P* < 0.05; **, *P* < 0.005. n.s., nonsignificant (error bars indicate SDs).

The membrane rearrangements that characterize macropinocytosis require activation of Rac1 and Cdc42 GTPases to support cytoskeletal reorganization. The drug amiloride analogue 5-(*N*-ethyl-*N*-isopropyl) amiloride (EIPA) reduces the submembranous pH, which is necessary for Rac1 and Cdc42 activation, and blocks macropinocytic fluid uptake (24). Treating A549-SLAMF1 cells with EIPA reduces both MeV-PP entry (Fig. 6A) and the number of infectious MeV foci formed (Fig. 6B). The specific macropinocytosis-inhibitory activity of EIPA was confirmed through targeted inhibition of PMA-induced dextran uptake in A549-SLAMF1 cells (Fig. 6C). Transfecting A549-SLAMF1 cells with GFP-tagged DN mutants of Rac1 and Cdc42 (N17) reduced MeV-PP entry (Fig. 6D). These constructs also significantly reduced MeV entry/infection (Fig. 6E). Interestingly, transient expression of GFP-tagged constitutively active (CA) (L61 for Rac1 and Cdc42) variants of these GTPases (Fig. 6D), as well as separate modulation of levels of p21-activated kinase-1 (PAK-1), a downstream effector of Rac1 and Cdc42 (23), using a CA mutant (Fig. 6F) or small interfering RNA (siRNA) knockdown (Fig. 6G) had only modest effects on MeV entry. These results show that although MeV entry is sensitive to EIPA, a traditional inhibitor of macropinocytosis, it does not appear to absolutely depend on the activity of Rac1 or Cdc42, especially since CA mutants were unable to boost entry. This contrasts with traditional requirements for macropinocytosis, suggesting perhaps a more complex entry pathway that may be specific to MeV-SLAMF1 interactions.

### Role for the mechanotransduction RhoA-ROCK-myosin II axis in MeV infection.

**FIG 7:**
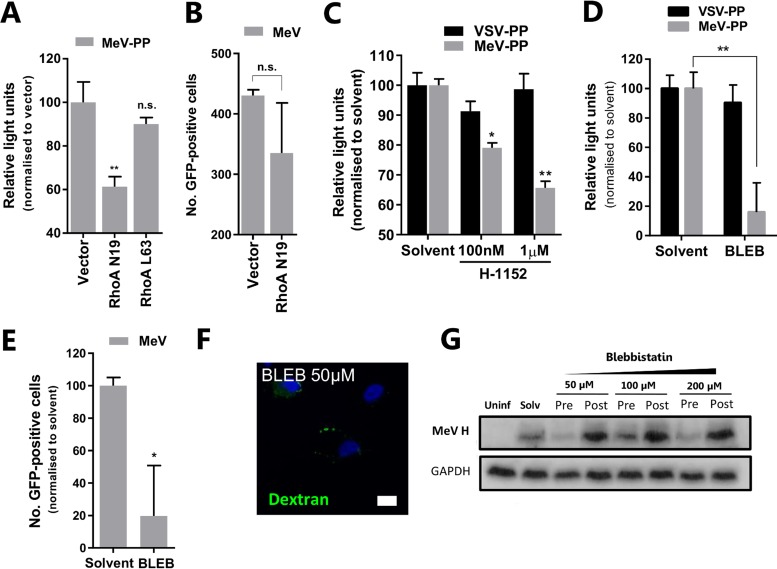
The RhoA-ROCK-myosin II axis is important for virus infection. (A) A549-SLAMF1 cells were transfected with constructs encoding myc-tagged DN (Rho N19) or GFP-tagged CA (Rho L63) of RhoA. Cells were infected with MeV-PP, and luciferase activity was measured 72 h later. (B) Similarly, cells were transfected with the same constructs, infected with MeV (MOI = 1), and incubated for 24 h; infected cells were quantified under UV microscopy. (C) A549-SLAMF1 cells were pretreated with the ROCKI inhibitor H-1152 at the indicated concentrations for 5 h before infection with MeV- or VSV-PP, and luciferase activity was measured 72 h later. (D) A549-SLAMF1 cells were pretreated with blebbistatin (BLEB) or solvent for 30 min before infection with MeV- or VSV-PP for 3 h, unbound virus was removed by washing, and cells were incubated for 72 h in complete medium prior to measurement of luciferase activity. (E) In a similar experiment, cells were pretreated with 100 μM BLEB for 30 min and infected with MeV (MOI = 1) for 1 h. Cells were trypsinized and incubated for 24 h before quantification of the frequency of GFP^+^ cells and total yield of virus. (F) A549-SLAM cells were incubated with blebbistatin at the indicated concentration for 30 min, washed, and incubated with 200 nM PMA in dextran-containing PBS for 30 min. Cells were then bleached, washed, and prepared for CSLM. Please refer to Fig. 6C for relevant control image. (G) To address if blebbistatin had a dose-dependent effect on MeV entry, A549-SLAMF1 cells were either pre- or posttreated with the drug at the indicated concentrations, trypsinized, and incubated at 37°C for 24 h. Total cell lysates were generated and resolved on a 15% SDS-PAGE gel prior to Western blotting using a polyclonal antibody raised specifically for MeV H cytoplasmic tail. Statistical analysis was performed using the Student *t* test. *, *P* < 0.05; **, *P* < 0.005. n.s., nonsignificant (error bars indicate SDs).

Filopodia and blebbing are characteristic features of migrating cells that rely on a dynamic actin cortex. Actomyosin contractility is regulated by intracellular signaling referred to as mechanotransduction. One of the best-understood examples is the ubiquitous RhoA-ROCKI-myosin II signaling axis, a feedback loop that responds to changes in extracellular matrix to modulate cellular motility (25). Given our earlier observations of MeV-induced changes in cellular morphology, we investigated a role for the RhoA-ROCKI-myosin II signaling axis in MeV entry. MeV-PP (Fig. 7A) and MeV (Fig. 7B) infection of A549-SLAMF1 cells was reduced by a DN variant (N19) of RhoA (another GTPase), although the latter was not significant. We attribute this muted effect, in part, to a combination of poor transfection efficiency in A549-SLAMF1 cells and the subsequent entry of MeV into nontransfected cells, where macropinocytosis remains active. However, in agreement with our previous experiments with RacI and Cdc42, the CA variant (L63) of RhoA had no significant effect on entry of MeV-PP (Fig. 7A) or MeV (data not shown). Interestingly, though, targeting the ROCKI protein, which acts downstream of RhoA to regulate myosin light chain phosphorylation, with the specific inhibitor H-1152 did significantly reduce MeV-PP entry (Fig. 7C). Nonetheless, the most notable effect on MeV entry was seen when targeting the myosin II element of the axis, a protein involved in the rapid contractility of the cortical actin network and essential for bleb formation. The myosin II-specific inhibitor blebbistatin significantly inhibited infection by MeV-PP (Fig. 7D) and MeV (Fig. 7E) but not VSV-PP. The specific inhibitory activity of blebbistatin was also confirmed through targeted inhibition of PMA-induced dextran uptake in A549-SLAMF1 cells (Fig. 7F). Blebbistatin was shown to inhibit MeV infection in a dose-dependent manner when target cells were treated before but not after infection (Fig. 7G), confirming a role for this compound in targeting steps involved in MeV entry. In summary, these data show that MeV entry is sensitive to perturbation of the mechanotransduction RhoA-ROCKI-myosin II axis and suggest a role for SLAMF1 to regulate cell motility and macropinocytic-like processes that are dependent on cytoskeletal reorganization.

### MeV induces cytoskeletal reorganization in a SLAMF1-dependent manner.

**FIG 8:**
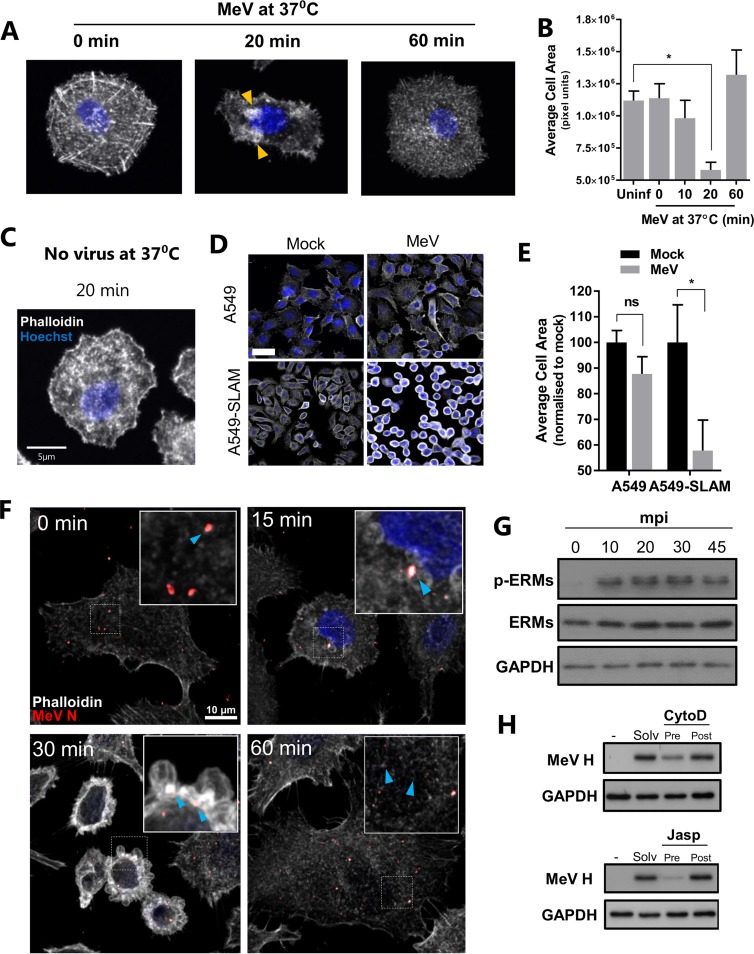
MeV induces cytoskeleton reorganization in a SLAMF1-dependent manner. (A and C) A549-SLAMF1 cells were synchronously infected with MeV (MOI, ∼15) for 1 h at 4°C (A) or incubated with 20% FBS-DMEM under the same conditions (No virus) (C). Cells were washed in cold PBS and incubated at 37°C for 0, 10, 20, or 60 min prior to fixation with 4% PFA, staining with phalloidin-TRITC (pseudocolored in white), and analysis by CSLM. Orange arrowheads indicate actin-enriched domains. (B) The average cell area (in pixel units) of individual cells was calculated based on representative micrographs of each condition using ImageJ. Statistical analysis was performed using the Student *t* test. *, *P* < 0.05 (error bars indicate SDs). (D) To assess the role of SLAMF1 in inducing the contraction of the cytoskeleton during MeV infection, A549 and A549-SLAMF1 cells were synchronously mock or MeV infected (MOI = 10), incubated at 37°C for 30 min, fixed, and analyzed by CSLM. The bar represents 30 μm. (E) Six representative micrographs of each condition were analyzed, and the average cell area was calculated. Statistical analysis was performed using a one-tailed Student *t* test. *, *P* < 0.05 (error bars indicate SDs). (F) A549-SLAMF1 cells were synchronously infected with MeV (MOI = 15), incubated at 37°C for 0, 15, 30, or 60 min, fixed, and prepared for CSLM. Blue arrowheads represent MeV N (in red) and actin (white) colocalization. (G) A549-SLAMF1 cells were synchronously infected with MeV (MOI, ∼15), washed, and incubated at 37°C for 0, 10, 20, 30, or 45 min prior to lysis and ERM phosphorylation analysis by SDS-PAGE and Western blotting. (H) A549-SLAMF1 cells were pre- or posttreated with the indicated concentrations of cytochalasin D (CytoD) or jasplakinolide (Jasp) and infected with MeV (MOI = 1). Total cell lysates were generated 24 h postinfection and resolved on a 15% SDS-PAGE gel prior to Western blotting using a polyclonal antibody raised against the cytoplasmic tail of MeV H.

The fluid uptake, filopodium formation, and blebbing observed during MeV infection of SLAMF1-positive cells are dependent on a highly dynamic actin cytoskeleton (23). Such morphological changes are associated with cell contraction and are a characteristic feature of macropinocytosis but not phagocytosis (23). Using phalloidin, a high-affinity probe for actin filaments, we demonstrated that MeV induces a transient cellular retraction peaking at 20 min, in association with actin bundle formation (Fig. 8A, orange arrowheads), before returning to baseline at 60 min postinfection (Fig. 8A). Quantification of cellular area after CSLM confirmed this retraction (Fig. 8B). Importantly, this retraction was not the result of incubating cells at 4°C (Fig. 8C) and, similar to dextran uptake, was dependent on SLAMF1 expression, observed both by CSLM (Fig. 8D) and through repeated quantification of cell area (Fig. 8E). To understand the role of these blebs in MeV internalization, we imaged particles and demonstrated a colocalization with actin-enriched membrane structures (Fig. 8F, 30 min, blue arrowheads). MeV particles were actin positive before internalization (Fig. 8F, 0 min), as previously reported (26). Again this association, together with the disruption of the actin cytoskeleton, was transient (Fig. 8F, 60 min). These data demonstrate that the contraction of the cell associates with virus-induced macropinocytic-like responses, i.e., blebbing, to facilitate viral entry in a receptor-dependent manner.

To examine the cellular response to this retraction, we focused on the ezrin-radixin-moesin family of proteins (ERMs). ERMs cross-link actin filaments and the plasma membrane and can regulate membrane blebbing; e.g., ezrin linkage inhibits blebbing through formation of the rigid uropod during directional migration (27). MeV infection of A549-SLAMF1 cells induced ERM phosphorylation within 10 min (Fig. 8G), and this was sustained for the duration of morphological changes, indicative of a cellular response to stabilize membrane-cytoskeleton interactions. To examine in detail whether a responsive and dynamic actin network is required for MeV entry, we used a panel of well-characterized chemical inhibitors. MeV infection was sensitive to both inhibition (cytochalasin D) and promotion (jasplakinolide) of actin polymerization (Fig. 8H) as well as inhibitors of the Arp2/3 complex (CK-666) and Wiskott-Aldrich syndrome protein (WASP; wiskostatin) (data not shown). Together, these results demonstrate that MeV-induced reorganization of the cytoskeleton, particularly the cortical actin network, is essential for infection and is regulated by ERM phosphorylation.

## DISCUSSION

**FIG 9:**
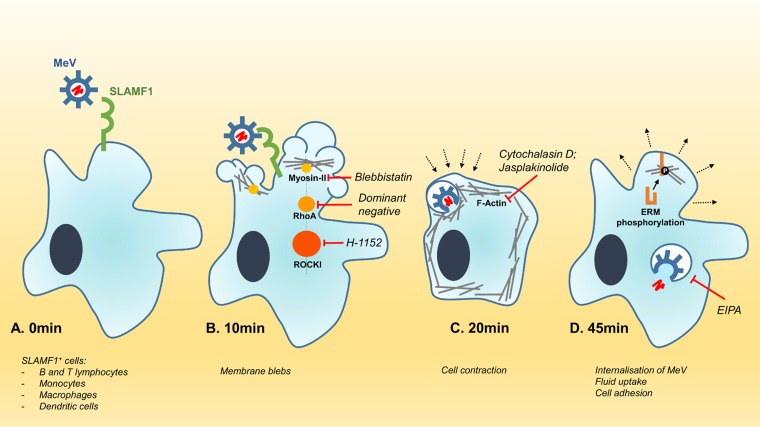
Model of endocytic MeV entry into SLAMF1-positive cells. (A) MeV binds to human SLAMF1 (CD150) via a specific protein-protein interaction between the viral hemagglutinin and SLAMF1. (B) Within 10 min, the receptor interaction induces the formation of membrane blebs, governed in part by the RhoA-ROCK-myosin II axis. Inhibition of this axis (with chemical inhibitors or dominant negative mutants) reduces MeV entry. (C) The formation of membranous blebs is followed by an acute retraction of the cell, orchestrated by the cortical cytoskeleton. Perturbation of this process with chemical inhibitors of actin modulation also reduces MeV entry. (D) The internalization of MeV particles is concurrent with fluid uptake via a macropinocytosis-like pathway, a process that is sensitive to chemical inhibition with EIPA. Within 45 min, infected cells begin to reestablish their characteristic morphology through specific phosphorylation of ERM proteins.

Until recently, paramyxovirus and pneumovirus genome entry into the cell was assumed to occur only at the cell surface after fusion of particle-associated glycoproteins with the plasma membrane and without the need for virus internalization (16, 28). The piece of data supporting this model is that triggering of many of their viral fusion proteins is pH independent, e.g., MeV F (29, 30). However, recent studies on entry of viruses with pH-neutral modes of fusion, e.g., RSV, Newcastle disease virus, Sendai virus, and Nipah virus, have demonstrated virions being endocytosed (17, 28, 31). Our data clearly show that MeV particles can internalize in a SLAMF1-dependent manner (Fig. 9). We propose that activation of the F protein is initiated at the cell surface and that this can prime either fusion and uncoating at the plasma membrane or an endocytic step involving particle internalization and concurrent genome escape. We believe that the pathway for MeV genome entry may therefore be determined by the valence of interactions between a virus particle and a host cell. When considering the factors involved in stimulating SLAMF1-dependent endocytic uptake, it is important to consider differences in the activity of non-particle-associated F-H complexes and the MeV particle itself, which is large (up to 1 μm), pleomorphic, and densely packed with viral glycoproteins. These may directly affect the interactions between the particle and the cell and, moreover, contribute to differing mechanisms of virus-cell and cell-cell fusion. This scenario is distinct from the case with RSV, for which proteolytic activation of F protein has to take place in the macropinosome before capsid uncoating and release of the genome (17). This step is also likely to be specific to mature particle entry during the vitally important first steps in human infection, since many of the late stages in MeV dissemination, particularly in the epithelia, rely on cell-cell contact and/or cell fusion (10). However, entry in this context is via a separate receptor, nectin-4 (8, 32). Importantly, MeV spread within the lymphatic system, in the early stages of disease, is SLAMF1 dependent, and this entry pathway may contribute to the widespread infection seen in lymph nodes (32).

The morphological response of cells to MeV infection is characteristic of the global membrane activation seen during classical macropinocytosis (23). This was supported by the significant uptake of extracellular fluid upon infection, sensitivity to EIPA, and the observation that incoming MeV particles associate with fluorescent dextran macropinosomes (Fig. 9D). Other viruses, e.g., RSV and vaccinia virus, reported to enter cells via macropinocytosis demonstrate similar phenotypes and are inhibited by these drugs (17, 21, 33). We also show that MeV is sensitive to perturbation of myosin II-associated blebbing through the specific inhibitor blebbistatin (Fig. 9B). However, we observed a differing sensitivity when examining a role for proteins such as Rac1, Cdc42, and PAK-1. These data are consistent with macropinocytosis not representing a single pathway but rather reflecting an umbrella term for several endocytosis mechanisms for fluid internalization with distinct, yet overlapping, phenotypes and mechanisms (34, 35).

Our studies show an integral role for SLAMF1 in MeV entry that most likely relates to a physiological role for this protein. Although SLAMF1 can regulate bacterial phagocytic killing, it seems unlikely that this pathway is involved in MeV entry, since SLAMF1^−/−^ cells are phagocytically competent (4). Moreover, the global membrane activation and fluid uptake seen following MeV infection are atypical of phagocytosis (23). The membrane blebs and filopodia formed are typical features of macropinocytosis and cellular motility required for responding to changes in the extracellular environment (36). It was interesting to see that MeV is reliant on the RhoA-ROCK-myosin II axis, a regulator of mechanotransduction (25). It will be interesting to examine whether the SLAMF1 cytoplasmic tail interacts with this axis and plays a role in immune cell migration. Recent findings that SLAMF1 interacts with beclin-1, which recruits a class III phosphatidylinositol 3-kinase that generates phosphatidylinositol-3-phosphate, support a role for this signaling molecule in membrane rearrangement and cell motility (4). Since immune cells are primary targets during MeV infection (6), it is possible that virus-induced SLAMF1-mediated signaling may influence subsequent immune cell interactions (37) and may contribute to the long-term immunosuppression associated with this infection.

MeV endocytosis appears to be reliant on the cell's cortical actin cytoskeleton and infection induced significant changes in host cell morphology (Fig. 9B and C). Similar cytoskeletal changes were reported following Kaposi's sarcoma-associated herpesvirus (KSHV), vaccinia virus, and African swine fever virus (ASFV) infections (21, 33, 38). Significantly, incoming MeV particles were found to associate with these cytoskeletal rearrangements; however, it remains to be determined whether the viral genome uses the cytoskeleton to traffic through the cell or to establish sites for transcription. In this context, it was interesting to observe rapid phosphorylation of the ERM proteins soon after entry, since moesin has previously been debated to be a MeV entry factor (39). A future area to investigate is the site of MeV internalization. While we observed colocalization of virus particles with membranous blebs, it is unclear if their collapse leads to virus internalization or whether the virus “surfs” on these structures to direct particles to specific sites for internalization (40). A recent review on virus entry highlights the role of immunoglobulin-like domains, similar to those in SLAMF1, to regulate pathogen entry (41). We speculate that MeV binding to SLAMF1 may induce receptor clustering as observed for the vaccine strain of MeV following the engagement of receptor CD46 (42), possibly explaining the extensive formation of filopodium-like structures we observed, although it should be noted that wild-type MeV does not bind CD46. MeV internalization may induce intracellular signaling via SLAMF1, and it will be interesting to examine whether receptor cross-linking is important, as reported for MeV interaction with CD46 (42).

In summary, these findings highlight a new internalization pathway for MeV to enter a host cell that is SLAMF1 dependent (Fig. 9). Given the ubiquitous usage of SLAMF1 as an entry receptor for all morbilliviruses (16), we believe that these findings will have wide-ranging impact for other members of this genus. Our observation that MeV engagement of SLAMF1 induces gross morphological changes and collapse of the cytoskeleton highlights a new physiological role for SLAMF1. This newly characterized pathway for MeV entry may provide a distinct advantage to the virus in certain immunological niches *in vivo*, i.e., during the establishment of infection in the respiratory tract or later in the draining lymph nodes of the head and neck. Research can now focus on examining whether morbillivirus infection perturbs SLAMF1-dependent immune function and explains virus-induced immunosuppression.

## MATERIALS AND METHODS

### Cell culture and viruses.

Cells of the A549 (ATCC CCL-185) and HEK293T (ATCC CRL-11268G-1) lines (from the ATCC) were engineered to express human SLAMF1 (43) and maintained in complete medium with puromycin (1 μg/ml; Sigma) at 37°C and 5% CO_2_. Parental A549 cells and HEK293T cells were grown in complete medium without puromycin. Vero cells (ATCC CCL-81) engineered to overexpress human SLAMF1 (Vero-SLAM [5]) were maintained in complete medium supplemented with Geneticin (0.4 mg/ml; Sigma). Epstein-Barr virus (EBV)-immortalized peripheral B lymphocytes (University of Birmingham cell collections) were cultured in complete medium. Recombinant MeV strain IC323 expressing the enhanced green fluorescent protein (EGFP) open reading frame (ORF) p(+)MV323EGFP was generated as previously reported by Hashimoto et al. (44). After initial rescue, MeV stocks were produced in Vero-SLAMF1 cells. After the development of substantial cytopathic effects, cells were freeze-thawed, and supernatants were clarified by centrifugation at 3,500 rpm for 30 min at 4°C, aliquoted, and stored at −80°C; this virus was used in experiments that did not require purified virus. The virus purification method was adapted from the method of Cathomen et al. (45). Vero-SLAMF1 cells, grown in eight T175 flasks to a confluence of approximately 40%, were infected with MeV (MOI, ∼0.1) in 8 ml of serum-free Dulbecco modified Eagle medium (DMEM) plus 1% penicillin/streptomycin (P/S) for 1 h at 37°C. This inoculum was then removed and replaced with 10 ml of complete medium. Three to 4 days later, flasks were vigorously shaken and supernatants were collected and clarified as before. Supernatants were layered onto a 20% sucrose cushion (wt/vol in Hanks' balanced salt solution [HBSS] with 25 mM HEPES NaOH [pH 7.4]) and centrifuged at 32,000 rpm for 3 h at 4°C in an SW32 Ti rotor (Beckman Coulter). The resultant pellets were resuspended in 1 ml of HBSS-HEPES, pooled, layered onto a 30-45-60% stepped sucrose gradient, and centrifuged at 35,000 rpm for 3 h at 4°C in an SW40 rotor (Beckman Coulter). Two opalescent bands were visualized under polarized light: one at the 60-45% interface (lower band) and the other below the 30-45% interface (upper band). The lower band was extracted using a needle and dialyzed separately overnight using a 10-kDa-molecular-mass-cutoff membrane tubing against 1 liter of HBSS-HEPES. Purified virus was then aliquoted, stored at −80°C, and titrated. The quality and purity of virus preparations were analyzed before use by SDS-PAGE and silver staining to confirm minimal cellular contaminants, as well as separate SDS-PAGE and Western blot analysis to confirm the presence of MeV proteins. Of note, only virus from the lower band was used in experiments, since it had a higher virus titer. Virus titers were calculated using the Reed-Muench method (46).

### Plasmids, cell transfections, and pharmacological inhibitors.

All plasmids used were transfected using TransIT-X2 dynamic delivery system (Mirus Bio LLC). The following plasmids were received as gifts: p8.91 and pCSFLW (Edward Wright (47); pEPS15-DN-RFP, pCav-1-DN-GFP, and pDyn2-DN-RFP (Joshua Rappoport [48]); pRhoA DN, pRhoA CA, pRac1 DN, pRac1 CA, Cdc42 DN, and Cdc42 CA (Patrick Caswell [49]); and, finally, pRLuc1-7 and pRLuc18-11 (Zene Matsuda [50]). Wild-type MeV Dublin strain (51) (a kind gift from Paul Duprex, Boston University) H and F protein ORFs were cloned into pcDNA3.1 after reverse transcriptase PCR (RT-PCR). The construct pDublin-HΔ24 encodes an MeV Dublin strain H truncated at the N terminus by 24 amino acids. It was amplified from the MeV Dublin strain H ORF using the forward primer 5′-AATGGATCCACCATGAACAGAGAACATCTTATGATT-3′, containing a BamHI restriction site, and reverse primer 5′-AATTGATATCCTACTATCTGCGRTTGGTTCCAT-3′, containing an EcoRV restriction site. The insert was digested and cloned into the BamHI/EcoRV restriction sites of pcDNA3.1. Similarly, the construct Dublin FΔ30 encodes a truncated version of the MeV Dublin strain F protein lacking 30 amino acids at the C terminus. The insert was amplified from MeV Dublin strain F ORF using the forward primer 5′-AATTGCTAGCACCATGGGTCTCAAGGCGAG-3′ and the reverse primer 5′-AATTGATATCCTACTACGCCCCCTGCAGCAACATATT-3′, containing the NheI and EcoRV restriction sites, respectively. The insert was digested and cloned into the NheI/EcoRV restriction sites of pcDNA3.1.

To generate the two constructs encoding PAK-1, mRNA was extracted from HEK293T cells using the GenElute mammalian total RNA miniprep kit (Sigma) according to manufacturer's guidelines. A cDNA library was generated from the extracted mRNA using Moloney murine leukemia virus reverse transcriptase (M-MLV RT; Promega) and a sequence of 15 thymine bases as a primer. cDNA encoding PAK-1 was amplified by PCR using the forward primer 5′-AATTGGATCCACCATGTCAAATAACGGCCTAGACA-3′, containing the BamHI restriction site (underlined), and the reverse primer 5′-AATTGCGGCCGCCTATTAGCTGCAGCAATCAGTG-3′, containing the NotI restriction site (underlined). Digested cDNA was inserted into pcDNA3.1, and the point mutation T423E was introduce by site-directed mutagenesis using the primer 5′-AGCAAACGGAGCGAGATGGTAGGAACC-3′ and respective reverse complement. All plasmids were sequenced prior to transfection. All pharmacological inhibitors—chloroquine, (−)-blebbistatin, phorbol 12-myristate 13-acetate (PMA), bafilomycin A1, 5-(*N*-ethyl-*N*-isopropyl)amiloride (EIPA), IPA-3, chlorpromazine (CPZ; Sigma-Aldrich), cytochalasin D, jasplakinolide, paclitaxel (originally named taxol; Calbiochem Merck Millipore), wiskostatin (Enzo Life Sciences), ML141, CK-666 (Tocris), and glycyl-H1152 (Cayman Chemical)—were tested for cytotoxicity prior to use by using a 3-(4,5-dimethylthiazol-2-yl)-2,5-diphenyltetrazolium bromide (MTT) assay to assess cell viability. Concentrations that caused a reduction in cell viability of more than 87% of the DMSO- or water-treated cells were not used.

### Pseudotyped virus particle genesis.

HEK293T cells were transfected with 3.5 μg each of pDublin-FΔ30 and pDublin-H Δ24 (encoding cytoplasmic tail truncations of MeV F protein and H) or 1 μg of pVSV-G, 1.5 μg of p8.91 (coding for HIV-1 gag-pol), and 1 μg of pCSFLW (luciferase reporter lentivirus backbone). Supernatants containing viral pseudotypes (PPs) were collected 48 and 72 h later, pooled, and clarified by centrifugation at 2,500 rpm at 4°C for 30 min. A549-SLAMF1 cells were seeded in a 96-well dish at 5 × 10^4^ per well. After 24 h, cells were pretreated with pharmacological inhibitors in fetal bovine serum (FBS)-free DMEM at the desired concentrations for 30 min at 37°C, followed by the addition of 100 μl of solution containing PPs, and then incubated for 5 h. Alternatively, A549-SLAMF1 or HEK293T-SLAMF1 cells were transfected with 1 μg of the desired constructs in 6-well dishes, and 48 h later, cells were transferred to 96-well plates. Cells were incubated with 100 μl of PPs for 5 h. In all cases, medium was replaced after 5 h, and following a further 72 h of incubation, lentiviral infection was assessed by lysing cells and measuring luciferase activity using the luciferase assay system (Promega) according to the guidelines of the manufacturer, on a Centro LB960 microplate luminometer (Berthold Technologies).

### Virus entry assay.

A549-SLAMF1 cells were pretreated or posttreated, in relation to infection, with pharmacological inhibitors of endocytic pathways. Pretreated cells were incubated with the drug for 30 min prior to addition of virus (MOI = 1) and incubated for an additional 1 h at 37°C in the presence of drug, while posttreated cells were inoculated for 1 h, washed, and incubated with drug for 37°C for 90 min. In both cases, cells were trypsinized to remove noninternalized virus and incubated for a further 24 h. Syncytia were counted under UV light, and whole-cell lysates were generated using Laemmli buffer and analyzed by Western blotting. To assess sensitivity to trypsin, A549-SLAMF1 cells were incubated with MeV (MOI = 30) at 4°C for 1 h. The inoculum was removed and cells were washed in cold phosphate-buffered saline (PBS) and incubated at 37°C for 0, 15, or 30 min. At these time points, cells were either trypsinized or detached with 2 mM EDTA in PBS (untreated) for 7 min at 37°C, pelleted, and analyzed by Western blotting. As a control, uninfected cells were treated in a similar way. To assess fusion upon entry mediated by the virus, we used a dual split reporter containing the Renilla luciferase gene (50). HEK293T-SLAMF1 cells were transfected with one part of the reporter, synchronously infected with purified MeV (MOI, ∼30), washed, and incubated at 37°C for 0, 15, 30, or 60 min prior to coculturing with HEK293T-SLAMF1 cells expressing the complementary part of the reporter. When testing the effect of chlorpromazine, cells were incubated for 30 min with 5 μg/ml prior to virus adsorption. The drug treatment was maintained throughout the short time course of the experiment. At each time point, cells were incubated for 2 h before luciferase activity was measured in a luminometer through the addition of 2 μg/ml of cell-permeative coelenterazine 400A (Biotium).

### Flow cytometry.

A549-SLAMF1 cells were either pre- or posttreated with 25 or 50 μM EIPA for 30 min and infected for 1 h at 37°C with recombinant MeV (MOI = 1) that expressed EGFP. After this period, cells were trypsinized, replated onto 6-well dishes, and incubated at 37°C for 6.5 h. Cells were detached using a 2 mM EDTA solution in PBS for 20 min, washed, and fixed in 4% paraformaldehyde (PFA). Cells were analyzed for EGFP fluorescence by flow cytometry (CyAn 642 TM ADP analyzer; Beckman Coulter).

### Western blotting.

All protein samples were generated using Laemmli buffer and reduced at 95°C for 5 min before analysis. Samples were resolved on 15% acrylamide gels, and blots were probed with primary antibodies, incubated in 5% milk-Tris-buffered saline (TBS) overnight, washed in TBS-Tween 20 (0.05%), and incubated with secondary antibodies coupled with horseradish peroxidase. Membranes were exposed to Clarity Western ECL substrate (Bio-Rad Laboratories) according to the manufacturer's guidelines and exposed to autoradiographic film.

### Phase-contrast and SEM.

Serum-starved A549-SLAMF1 cells or B-lymphoblastoid cells were prechilled on ice and MeV (MOI, ∼45) bound to the cells at 4°C for 1 h. Uninfected cells were treated with DMEM plus 20% FBS. After 1 h, the inoculum was removed and cells were washed in ice-cold PBS before being moved to 37°C for 10, 20, or 60 min. For phase-contrast microscopy, cells were fixed with 4% PFA and visualized by phase-contrast microscopy using an inverted UV microscope (Nikon Eclipse TE2000-5 microscope coupled with a Nikon HB-10101AF superhigh-pressure mercury lamp) equipped with a Hamamatsu C472-95 digital camera). Ten micrographs for each condition were recorded, and the percentage of cells presenting membrane blebs was determined in relation to the total cell count per field of view. For scanning electron microscopy (SEM), cells were fixed overnight with 4% PFA and 2.5% glutaraldehyde at 4°C. Fixed cells were washed in PBS, postfixed in 2% osmium tetroxide, dehydrated in acetone, and critical-point dried for 2 h. Finally, cells were coated with a 3-nm layer of platinum and visualized by SEM (JSM-7000F JEOL SEM; Oxford Instruments INCA EDS system). For quantification of blebbing and ruffling cells, 12 micrographs of each sample were blinded, randomized, and scored.

### Immunofluorescence assays.

Cells were plated onto glass coverslips and synchronously infected with MeV at 4°C for 1 h with gentle rocking. As an uninfected control, cells were incubated with medium containing 20% FBS. Cells were washed and incubated in PBS at 37°C for the desired times or, in the fluid uptake assay, incubated in PBS containing 0.25 mg/ml of dextran-Alexa Fluor 488 (molecular weight, 10,000; Molecular Probes 627; Life Technologies). Surface-bound dextran was bleached by incubating with cold bleach buffer (10 mM sodium acetate, 50 mM NaCl [pH 5.5]) for 10 min before washing with PBS. In all cases, cells were fixed in 4% PFA for 10 min at room temperature, permeabilized with 0.1% Triton X-100 in PBS for 10 min, and blocked with 1% bovine serum albumin (BSA)–PBS for 30 min at room temperature. Cells were incubated with primary antibodies in 1% BSA at 4°C overnight, washed in PBS, and incubated with secondary antibodies conjugated with Alexa Fluor dyes (Invitrogen) for 1 h at room temperature. For staining of actin, fixed cells were incubated with a solution of 0.5 μg/ml of the phalloidin conjugate tetramethylrhodamine B isocyanate (phalloidin-TRITC; Sigma-Aldrich) in PBS for 30 min at room temperature and washed in PBS. Slides were mounted using Hoechst 33342 in Mowiol 632 mounting medium (Merck Millipore). To visualize the cells, we used a Leiss LSM 510 Meta confocal microscope, and further analysis was performed using ZEN software and ImageJ.

### Antibodies.

Anti-cytoplasmic tail of measles virus hemagglutinin and anti-measles virus nucleocapsid 505 were gifts from Roberto Cattaneo's lab (45). Anti-glyceraldehyde-3-phosphate dehydrogenase, anti-phospho-ezrin (Thr567)/radixin (Thr564)/moesin (Thr558) (41A3; monoclonal antibody [MAb] 5175), anti-ezrin/radixin/moesin (3142), anti-alpha-p21-acivated kinase, and anti-rabbit IgG1 coupled with horseradish peroxidase were all purchased from Cell Signaling Technology.

### Image analysis.

To calculate total cell fluorescence in the fluid phase uptake experiment, micrographs recorded at 488 nm were converted to 8-bit images. A total of 16 cells per sample were individually selected, and area and mean fluorescence intensity were calculated using ImageJ. For each cell, 4 different regions were selected and the corresponding background mean fluorescence was subtracted from that cell intensity to generate corrected cell fluorescence. The average area of phalloidin-stained cells was calculated by bringing the background of individual micrographs to a level of threshold (overflow) that enabled clear distinction of the cell's edges and measurement of area (pixel units). Confocal intensity profiles of dextran and MeV N-positive cells were obtained by calculating red and green channels intensities across the indicated lines, using ZEN microscope and imaging software (Zeiss 2.3, blue edition).

### Statistical analysis.

Statistical analysis was performed using GraphPad Prism 5 where groups of two were analyzed using the unpaired two-tailed Student *t* test, unless otherwise stated. We considered a *P* value lower than or equal to 0.05 as a minimum threshold for significance.
